# Precursors and Effects of Self-reported Parental Reflective Functioning: Links to Parental Attachment Representations and Behavioral Sensitivity

**DOI:** 10.1007/s10578-023-01654-2

**Published:** 2024-02-07

**Authors:** Melanie T. Kungl, Sandra Gabler, Lars O. White, Gottfried Spangler, Pascal Vrticka

**Affiliations:** 1 Department of Developmental Psychology, Friedrich-Alexander-Universität Erlangen-Nürnberg, Naegelsbachstrasse 49a, 91052 Erlangen, Germany; 2 Department of Child and Adolescent Psychiatry, Psychotherapy, and Psychosomatics, University of Leipzig, Liebigstraße 18, 04103 Leipzig, Germany; 3 Berlin Psychological University, Am Köllnischen Park 2, 10179 Berlin, Germany; 4 Department of Psychology, Centre for Brain Science, University of Essex, Wivenhoe Park, Colchester, CO4 3SQ UK; 5 Max Planck Institute for Human Cognitive and Brain Sciences, Stephanstraße 1A, 04103 Leipzig, Germany

**Keywords:** Parental reflective functioning, Sensitivity, Attachment, PRFQ, Parent–child interaction

## Abstract

Parental reflective functioning is thought to provide a missing link between caregivers’ own attachment histories and their ensuing parenting behaviors. The current study sought to extend research on this association involving 115 parents, both mothers and fathers, of 5-to-6-year-old preschoolers using the German version of the Parental Reflective Functioning Questionnaire (PRFQ). Our study was the first to combine Adult Attachment Interview classifications of parental attachment, behavioral observations of parental sensitivity and PRFQ ratings while drawing on a sizable father subsample. We found theoretically consistent significant relations between all measures, while our results particularly highlighted the role of dismissing attachment for decreases in parenting quality on both cognitive and behavioral levels as the dismissing status differentially affected specific components of self-reported parental reflective functioning and observed sensitivity. Interestingly, these patterns were largely comparable in mothers and fathers. Exploratory mediation analyses further suggested that decreased parental reflective functioning may partially mediate the relationship between parents’ dismissing attachment and decreased parental sensitivity. Thus, for prevention and intervention programs targeting parental sensitivity and thus children’s long term healthy mental development, the interplay between parental reflective functioning and parents’ own attachment history emerges as a key mechanism. Finally, our study served as a further validation of the PRFQ given the caveat that the pre-mentalizing subscale may need further revision in the German version.

**Supplementary Information:**

The online version contains supplementary material available at 10.1007/s10578-023-01654-2.

## Introduction

The overarching construct of reflective functioning as postulated by Fonagy et al. [1] describes a manifestation of an individual’s mentalizing capacity [2]. Within this context, mentalizing is seen as “the capacity to understand one’s own and others’ behavior in terms of underlying mental states and intentions, and more broadly as a crucial human capacity that is intrinsic to affect regulation and productive social relationships” [2], p. 269]. It therefore represents a key ability to navigate the social world [3] and enables dyadic emotion regulation. More specifically, applied to the context of parenting and the parent–child dyad, parental reflective functioning refers to parents’ mentalizing abilities in the relationship with their children. Parents with high levels of parental reflective functioning can reflect upon their children’s mental experiences, giving meaning to children’s behavior, as well as their own experiences as caregivers [2]. This further encapsulates parents’ ability to hold their children’s mental states in mind and treat children as psychological agents [2, 3].

In turn, parental reflective functioning is assumed to positively affect children’s healthy mental development by supporting them in gaining access to their own emotions and intentions and making sense of their own and others’ behaviors. For example, prior evidence clearly supports a key role of parental reflective functioning in the development of children’s attachment security, which in turn has repeatedly found to be crucial for children’s social-emotional development and psychosocial adjustment [4, 5]. Parental reflective functioning may facilitate this development potentially by virtue of its impact on parental responsiveness to their offspring’s affective needs [6–8]. Given this crucial link between parental reflective functioning, sensitive parenting and child adjustment, it seems important for research and practice to further explore the relation between parental reflective functioning and sensitive parenting as well as its possible antecedents. The current study importantly expands existing literature on correlates of self-reported parental reflective functioning by applying a multi-modal approach using gold-standard narrative measures of parental attachment and observational methods of parental sensitivity.

In the current literature, a variety of measures to examine parental reflective functioning are mentioned. In fact, Schiborr et al. [9] identified 15 instruments to assess child-focused parental mentalizing. Operationalizations of parental reflective functioning range from traditional narrative measures—such as the Mini-Parent Reflective Functioning Interview (Mini-PRFI, [10]) and the Parent Development Interview (PDI, [11])—to a more recently developed self-report measure, the Parental Reflective Functioning Questionnaire (PRFQ) by Luyten et al. [3]. While interview-based narrative measures are typically considered the “gold standard”, these measures are very time consuming and require a fair amount of human and financial resources. In response to the need for more rapid and economical assessment of parental reflective functioning, the PRFQ was designed as a brief screening tool for larger samples, including parents from a wide range of educational backgrounds [12]. Anis et al. [13] have linked PRFQ subscales to narrative (parental) reflective functioning measures, however, no study to date has examined the link between the PRFQ and interview-based parental attachment. The present research thus is important as it still remains unclear whether different operationalizations of parental reflective functioning may tap into different aspects of the broader construct [12, 14, 15].

The PRFQ comprises three subscales corresponding to the key components of parental reflective functioning: interest and curiosity in mental states, certainty about mental states, and pre-mentalizing modes [3]. The first subscale (interest and curiosity) describes whether parents are interested in finding out what their child’s inner world looks like and in understanding their child’s internal states, emotions and intentions. The second component (certainty) refers to parents’ acceptance of a certain opacity of their child’s mental state with high scores on this scale illustrating a tendency to possibly judge too hastily about it. The latter component (pre-mentalizing modes) refers to parents’ ability to enter and adequately reflect upon the child’s internal world with high scores representing parents’ tendency to make maladaptive attributions about the child’s motives and intentions [16]. All three components differently relate to certain aspects of parenting. For example, increased maternal interest and curiosity predicted increased tolerance of distress in a baby simulator paradigm [17]. In addition, mothers with high levels of interest and curiosity reported more emotional awareness, while higher levels of pre-mentalizing were positively associated with deficits in mothers’ emotion regulation [18, 19].

The PRFQ’s convergent validity was determined by Anis et al. [13] who found PRFQ scores to significantly relate to reflective functioning rated from the PDI. A few validation studies furthermore found the PRFQ to be associated with parent self-reports of adult romantic attachment, parenting behavior and evaluations of the parent–child relationship [3].

In our study, we aimed to explore the links between parental reflective functioning derived from the PRFQ, attachment representations and sensitivity in parents of preschool children beyond self-reports. Our study was the first to apply a multi-modal approach encompassing narrative and behavioral measures. Second, we aimed to contribute to the current PRFQ literature by obtaining more information about the questionnaire’s validity and reliability.

## Associations Between Parental Reflective Functioning and Parental Attachment

Within contemporary attachment research, parental reflective functioning is assumed to constitute one possible link bridging the transmission gap between parental attachment representation and child attachment [20]. Prior evidence suggests that parents with secure attachment representations regarding their own caregiving experiences show an increased interest in their children’s inner world and are more capable to adequately reflect upon it. For example, using narrative measures of parental reflective functioning derived from the PDI, Slade et al. [20] found that mothers who were classified as secure (versus insecure) in the Adult Attachment Interview (AAI, [21]) scored higher on parental reflective functioning. Further insight comes from a study on parental mind-mindedness, a construct closely associated with parental reflective functioning [22]. Here, parents classified as secure (versus insecure/unresolved) in the AAI before the birth of their child also showed increased infant-related mind mindedness during free play [23].

Similarly, using the PRFQ and self-reports of attachment dimensions, the PRFQ subscale pre-mentalizing was repeatedly found to positively associate with attachment anxiety and avoidance in the Experiences in Close Relationships Questionnaire (ECR-R, [3, 16, 18, 24]). This PRFQ subscale furthermore appears to mediate (and indirectly affect) the relationship between insecure parental attachment and different domains of parenting stress in first-time parents [16]. Increased pre-mentalizing was also related to more authoritarian parenting styles [18] and was found to be an intervening variable in the relationship between insecure parental attachment and children’s behavior problems [25]. Beyond the high and positive correlation between pre-mentalizing and self-reported attachment avoidance and anxiety that Luyten et al. [3] identified in their validation study, the authors also reported moderate negative associations between certainty about mental states and attachment avoidance as well as anxiety. However, both insecure attachment dimensions were unrelated to interest and curiosity. In contrast, interest and curiosity negatively related to attachment avoidance [18] and positively to attachment security using a different self-report of attachment, namely the Attachment Style Questionnaire (ASQ, [26, 27]).

While the strength of former studies on parental attachment and the PRFQ lie in large sample sizes including participants from a variety of backgrounds, results need further validation. Given that self-reports of attachment as well as parental reflective functioning were completed by the same informants, a shared method bias cannot be excluded. Also, while the AAI is considered the gold-standard to measure adult attachment, association strengths between narrative measures and self-reports of attachment are rather weak [28]. In addition, from a theoretical perspective, attachment in the AAI should be more reliably related to parental reflective functioning as it captures parents’ own caregiving experiences, which contrasts markedly with emotions in adult relationships that are subject to most self-report measures (i.e., the ECR-R). Our current study thus addressed the gap in the literature on associations between the PRFQ and gold-standard measures of attachment, i.e., the AAI.

## Associations Between Parental Reflective Functioning and Parental Sensitivity

Considering Ainsworth’s definition of sensitivity (i.e., perception, correct interpretation as well as prompt and adequate response to children’s signals; [29]), parental reflective functioning appears to be a precondition for sensitive parenting, which in turn is highly relevant to children’s healthy mental development. Parental reflective functioning facilitates understanding children’s mental states and intentions that is crucial for responding to them in a sensitive way. Thus, higher levels of parental reflective functioning should be associated with parents’ increased sensitivity to children’s emotional signals and therefore appropriate dyadic emotion regulation. Using the Mini-PRFI, lower levels of parental reflective functioning were linked to observed parental insensitivity, which mediated the relationship between parental reflective functioning and infant attachment organization in the strange situation [30]. Furthermore, disruption in observed mother-infant affective communication was inversely related to levels of parental reflective functioning in the PDI [6]. Also, the relationship between parental mentalizing and parental sensitivity was confirmed in meta-analyses [8, 31]. However, a recent review by Stuhrmann et al. [31] identified only one study that linked the PRFQ to observed parental sensitivity. Notably, this study only included a sample of mothers with their infants and assessed sensitivity using a still-face paradigm [32], while studies including more complex interactions like parent–child problem solving are missing. Furthermore, only two of the studies reviewed by Stuhrmann and colleagues included fathers, which further underlines the relevance of our current study.

Using the PRFQ, Rutherford et al. [33] found expected associations between self-reported parental reflective functioning in the PRFQ and parental neural correlates of infant affective cue perception. Also, lower levels of parental reflective functioning (i.e., increased pre-mentalizing modes and increased certainty about mental states) derived from the PRFQ were found to be associated with less self-reported caregiver emotional availability [3]. In contrast, increased self-reported curiosity in mental states (indicating higher parental reflective functioning) was positively correlated with caregivers’ emotional availability in the same study. In a sample of mothers with postpartum depression and their infants, higher levels of self-reported pre-mentalizing from the PRFQ (indicating lower levels of mentalizing) were associated with greater decreases in maternal sensitivity during the still face paradigm, whereas no correlations between changes in maternal sensitivity and the other PRFQ subcales interest and curiosity and certainty about mental states were found [32]. In our study, we will add to this prior evidence associating the PRFQ with behaviorally coded aspects of parental sensitivity observing parents and their pre-school children during a problem-solving task.

Furthermore, we explored the interplay between all relevant constructs combining the three variables parental attachment, parental reflective functioning and parental sensitivity into one study. Most recently, a study by Dollberg [34] followed a similar lead exploring links between self-reported maternal attachment dimensions (via the ECR), maternal mentalizing (via the PDI and observed mind-mindedness postpartum) as well as maternal sensitivity coded behaviorally during free play. Maternal attachment anxiety was related to poorer maternal mentalizing in terms of mind-mindedness, but also to somewhat increased parental reflective functioning from the PDI. Increased mind-mindedness during free play but not parental reflective functioning from the PDI was associated with higher levels of maternal sensitivity. Still, we do not know of any study to date that has examined these links beyond infancy, and no study that has investigated the links between parental reflective functioning in the PRFQ, parental attachment representations in the AAI and observed parenting behavior.

## Study Aim/Research Questions

Up to now, studies linking parental reflective functioning in the PRFQ to parental attachment and sensitivity are restricted to those solely using self-report data. Also, we are not aware of any study that has addressed the interplay between these three variables in preschool children. In general, the majority of studies on parental reflective functioning (including different measures) focusses on mothers, especially during the early months of parenting. Regarding the German version of the PRFQ, to our knowledge, there is only one study including a clinical sample of mothers and their infants [32]. The current study therefore crucially extends research on parental reflective functioning to both mothers and fathers of pre-school children. Thereby, our major aim was to investigate associations between parental reflective functioning, attachment, and sensitivity, with the multi-modal approach being an outstanding value of our study.

We expected to replicate the widely established link between attachment representations and parental sensitivity [35] in our preschool sample. Similarly, links between parental reflective functioning and parenting behavior are mostly limited to infant studies and/or studies using self-reports of parental behavior (e.g., [3, 6, 31]). Thus, we investigated whether parents showing increased scores in the PRFQ would score higher on different aspects of parental sensitivity when interacting with their 5-to-6-year-olds. In line with the outlined literature finding associations between interview-based assessments of attachment and parental reflective functioning [20] and among self-report measures [3, 16] we expected secure parental attachment in the AAI to be associated with higher parental reflective functioning in the PRFQ. Notably, given the non-longitudinal design of the current study, we further only exploratively aimed to test PRFQ scores as a mediator between parental attachment and parental sensitivity.

Based on previous findings, our hypotheses were:

- H1: Parental secure attachment representations are associated with higher levels of parental reflective functioning
- H2: Parental secure attachment representations are associated with higher levels of observed parental sensitivity
- H3: Higher levels of parental reflective functioning are associated with higher levels of observed parental sensitivity
- H4: Parental reflective functioning mediates the association between parental attachment representations and parental sensitivity

Finally, as we examined mother–child as well as father–child dyads, we exploratorily investigated possible gender-differences in parental reflective functioning, attachment and sensitivity.

## Methods

### Participants and Study Design

Data was acquired within a multi-modal study combining neuroscientific methods (functional near-infrared spectroscopy—fNIRS—hyperscanning and functional magnetic resonance imaging—fMRI) with behavioral assessments and narrative measures to investigate different aspects of parent–child relationships. Parental attachment and parental sensitivity were assessed at two separate laboratory visits, while self-reports of demographic data and parental reflective functioning were completed online as part of a test battery realized using SoSci Survey [36]. Data collection took place between May 2018 and March 2020.

Our sample consisted of 115 parent–child dyads that were recruited from a database of volunteers based in a mid-size city in Germany. Due to a neuroscientific assessment that was part of the larger project this study was situated in, inclusion criteria included being right-handed, physically healthy, and not having any history of psychiatric illness. Parents (59 mothers) were aged 28.5 to 49.3 years (*M* = 38.06, *SD* = 4.70) with fathers (*M* = 39.03, *SD* = 4.89) being slightly older than mothers (*M* = 37.15, *SD* = 4.36), *p* = 0.032, *F* (1,113) = 4.716. All children (56 female) were 5-to-6 years of age (*M* = 5.35, *SD* = 0.27) by the time of the behavioral assessment and biologically related to the participating parent. Child biological sex was counterbalanced across mother- and father–child dyads. All dyads were of White European origin. Family income was < 3000 Euro/month in 24.3%, 3000–5000 Euro/month in 44.3%, and > 5000 Euro/month in 25.2% of families (6.1% decided not to answer the question). 61% of mothers and 51.8% of fathers held a university degree.

Eleven more parent–child dyads took part in the parent–child interaction and were administered the AAI but were excluded as data was incomplete due to technical problems during the interview (*n* = 2) or missing questionnaires (*n* = 9). All parents provided written informed consent for themselves and their children and were compensated for participation, while children received a small gift. The study was conducted following ethical principles in line with the Declaration of Helsinki and was approved by the local ethics committee.

## Measures

### Parental Reflective Functioning

We assessed parental reflective functioning using the German version of the PRFQ [3]. The PRFQ is a brief self-report measure consisting of 18 items that covers three key domains of parental reflective functioning, i.e., pre-mentalizing modes, certainty about mental states, and interest and curiosity in mental states. Parents are asked to rate each statement on a 7-point Likert scale ranging from 1 (strongly disagree) to 7 (strongly agree). Importantly, due to the complexity of parental reflective functioning, the PRFQ does not yield a single score but three separate scores, one for each subscale. The first subscale, pre-mentalizing modes, refers to parents’ incapability of mentalizing regarding their child. It comprises items such as “My child cries to embarrass me” and high scores on this scale are indicative of an increased struggle to understand why their child behaves in certain ways. The second subscale, certainty about mental states, refers to the parents’ acknowledgement about the opacity of their child’s mental state, which is a central component of parental reflective functioning. Thus, parents who are not aware that mental states are often not readily inferable will score high on this scale consisting of items such as “I always know what my child wants”. Conversely, parents scoring very low on this scale may be highly confused and uncertain about their child’s mental state. The third subscale, consisting of items such as “I am often curious to find out how my child feels”, reflects parents’ interest and curiosity about their child’s mental states. Parents scoring high on this scale usually try to take their child’s perspective and are eager to find out about their child’s inner world to better understand their behavior.

Internal consistencies (Cronbach’s alpha) for each subscale were reported by Luyten and colleagues [3] as follows: 0.70, 0.82 and 0.75 for pre-mentalizing modes, certainty about mental states and interest and curiosity, respectively. The PRFQ was found to be related to self-reported parental attachment dimensions (as assessed by questionnaires), emotional availability and parenting stress in generally theoretical explicable ways (see [3]). Only recently, Carlone et al. [12] reported the PRFQ’s good to excellent test–retest reliability across 1 year as well as it’s convergent validity comparing the PRFQ to task-based measures of mentalization. Anis et al. also found associations between the PRFQ and parental reflective functioning scored from interviews (i.e., the PDI), and confirmed construct validity especially for the PRFQ subscales certainty about mental states as well as interest and curiosity [13], but see [12].

Using the German version of the PRFQ in our study Cronbach’s alpha was 0.44, 0.82 and 0.63 for pre-mentalizing modes, certainty about mental states and interest and curiosity respectively, indicating very low reliability of the pre-mentalizing modes subscale, which has also been reported by [32]. A factor analysis confirmed the 3-factor solution of the PRFQ, with all items except for one loading on the expected factor (PRFQ internal validity and factor structure are discussed in the limitation section).

Using Spearman’s Rho, we found that the pre-mentalizing modes subscale was not correlated with certainty about mental states, *r* = 0.26, *p* = ns, or interest and curiosity, *r* = − 0.13, *p* = ns. Certainty about mental states and interest and curiosity were positively correlated, *r* = 0.22, *p* = 0.019.

### Parental Attachment Representations

To assess parents’ attachment representations we used the AAI [21]. The AAI is a widely used complex semi-structured interview with high reliability and validity (for a review see [37]). It consists of 18 questions that target the evaluation of parents’ early experiences with their own primary caregivers (mostly both parents) in childhood as well as experiences of separation and significant loss. It further asks individuals to reflect on how the relationship with their primary caregivers has changed over the years and what they have learned from their experiences, especially regarding their own parental role. In the present study, we used the German translation of the AAI protocol [38]. Post-graduate students conducted the interviews after receiving extensive training. Interviews were transcribed verbatim and then coded using the scoring and classification manual provided by Main et al. [39]. In accordance with the manual, individuals were ascribed one of the three organized attachment categories, namely secure, insecure-dismissing or insecure-preoccupied after evaluating the transcripts regarding narrative coherence, idealization, and derogation of attachment as well as current anger and passivity of speech. Coding was done by two certified coders that have already reached good agreement in previous studies. Coders further double coded ten randomly selected transcripts from our study and reached 80% agreement regarding the three-way classification.

### Parental Sensitivity

#### Behavioral Assessment

We assessed parental sensitivity in a semi-structured observation during the fNIRS hyperscanning part of the overall multi-modal study. In the testing room, the parent–child dyad was seated face to face at a table and was guided through a cooperative and an individual problem-solving condition (2 × 2 min each) consisting of solving tangram puzzles. The experimental design was used for comparing interpersonal neural synchrony within the parent–child dyad across both conditions (see [40]). The tangram task episodes were followed by a 6-min episode during which parents were instructed to guide the child through a preschool-sheet. The whole procedure was videotaped. In our study we scored parental sensitivity during both cooperative problem-solving conditions (tangram task) and the preschool-sheet task leading to a total of 10-min of observation time.

#### Behavioral Coding

We coded parental sensitivity from videos using the NICHD Study of Early Child Care Parent–Child Interaction Rating Scales [41] for preschool-children (36–54 months) provided by Owen et al. [42]. The scales cover multiple dimensions of parental sensitivity. In our study we used four out of the five 7-point Parent Scales, i.e., supportive presence, respect for child’s autonomy, stimulation of cognitive development and quality of assistance. Scoring was also done on an additional scale (hostility), but the scale was excluded due to a lack of occurrence of hostile behavior above the low range.

Supportive presence reflects the extent to which the efforts of the child are emotionally supported by the parent. The second scale, respect for the child’s autonomy, describes the acknowledgement of the child’s individuality, motives, and perspective by the parent. Stimulation of cognitive development refers to the degree to which the parent tries to foster the child’s cognitive and mental development and stimulate a higher level of understanding. Finally, the quality of assistance scale assesses how well the parent structures the situation for the child by, for example, assisting the child with the provision of logical steps.

We rated each of the scales separately for the tangram task and the preschool sheet task and averaged scores subsequently. Three post-graduate psychology students who received extensive training by the first authors coded the videos. They were all blind to other data and trained until satisfying levels of reliability were achieved in all scales before coding videos from our study (weighted kappa: 0.65 to 0.83). Post-hoc calculated inter-rater reliability of 17 video tapes was 0.72 for supportive presence, 0.65 for respect for child’s autonomy, 0.76 for stimulation of cognitive development, and 0.73 for quality of assistance (weighted kappa).

### Statistical Analyses Plan

Prior to our analyses, we examined variable distribution. This revealed that the pre-mentalizing subscale was positively skewed (0.995). To restore the data to normality we conducted a log transformation for this scale before computing any analyses of variance.

To investigate the relationship between parental attachment and a) parental reflective functioning and b) parental sensitivity during standardized problem-solving tasks in mothers and fathers, we conducted two separate multivariate analyses of variance (MANOVAs) with attachment classification as the independent variable**.** We entered the three PRFQ scales and the four sensitivity scales respectively, as the dependent variables. To test possible differences between mothers and fathers, we added parental gender as another independent variable in both MANOVAs. Where parental age was correlated with the outcome, we included it as a covariate. Child gender was not included as it was not related to either PRFQ or parental sensitivity. Effect sizes were indicated as partial η^2^. When multivariate effects of attachment were significant, we looked at univariate tests of between-subject effects. For all analyses of variance, Box’s Test of Equality of Covariance Matrices and Levene’s Test of Equality of Error Variances were not significant. For post-hoc pairwise comparisons, we applied the False Discovery Rate (FDR) correction.

Further, to investigate the predictive value of parental reflective functioning on parental sensitivity and effects of parental gender on this relationship, we performed multiple hierarchical regression analyses for each sensitivity scale. We included parental gender in step 1, the three subscales of parental reflective functioning in step 2 and finally the interaction terms of the three subscales and parental gender in step 3 of the model to test interaction effects on parental sensitivity. We coded parental gender with − 1 and + 1 as recommended for dichotomous variables in regression models [43, 44]. Where parental age was correlated with the outcome, it was entered in step 1 of the regression analyses followed by the other variables in the order described above.

## Results

### Descriptive Data

#### Parental Attachment Classifications

Regarding the organized attachment state-of-mind in our sample, we classified 63 parents (34 mothers) as secure, followed by 33 parents (12 mothers) classified as insecure-dismissing and 19 parents (13 mothers) classified as insecure-preoccupied. This distribution is comparable to other non-clinical European samples as suggested by meta-analytic data [45]. On a marginally significant level, the dismissing classification was slightly over-represented in fathers while more mothers than fathers were classified as preoccupied, main effect of attachment classification group *X*^*2*^ (2, *N* = 115) = 5.36, *p* = 0.069. The distribution of attachment classifications was independent of parental age.

#### Parental Reflective Functioning Scales

Table 1 depicts means and standard deviations of the three PRFQ scales in the total sample and by parent gender. Univariate analysis of variance showed that there were no differences between fathers and mothers with respect to the pre-mentalizing modes and certainty about mental states subscales, however, mothers scored significantly higher than fathers with respect to the interest and curiosity subscale, *F* (1,113) = 6.09, *p* = 0.015. Parental age was unrelated to parental reflective functioning.

**Table 1:** Minimum and maximum scores, means and standard deviations of PRFQ scales and parental sensitivity scales in the total sample and by parent gender

	Total sample				Mothers (*n* = 59)		Fathers (*n* = 56)	
	Min	Max	*M*	*SD*	*M*	*SD*	*M*	*SD*
*Parental reflective functioning*								
Pre-mentalizing modes	1	3.83	1.81	0.59	1.76	0.62	1.86	0.56
Certainty about mental states	1.33	6.50	4.11	1.12	4.09	1.09	4.13	1.15
Interest and curiosity	3.17	7.00	5.20	0.83	5.39	0.85	5.01	0.78
*Parental sensitivity*								
Supportive presence	1	7	3.75	1.37	3.95	1.37	3.54	1.35
Respect for autonomy	1	7	3.89	1.15	4.05	1.18	3.71	1.09
Stimulation of cognitive development	1	7	3.30	1.11	3.47	1.04	3.13	1.16
Quality of assistance	2	7	4.55	1.18	4.78	1.08	4.30	1.24

*N* = 115, *M* = mean, *SD* = standard deviation

#### Parental Sensitivity Scales

Means and standard deviations for the four parental sensitivity scales for the total sample as well as by parental gender are presented in Table 1. Univariate analyses of variance revealed that mothers scored significantly higher than fathers with regard to parents’ quality of assistance, *F* (1,113) = 4.84, *p* = 0.030, while the difference between mothers and fathers regarding respect for autonomy only marginally approached significance, *F* (1,113) = 2 0.90, *p* = 0.091. Parental age was solely significantly negatively related to stimulation of cognitive development, *r* = − 0.19, *p* = 0.037.

Table 2 descriptively shows the relations between parental sensitivity and parental reflective functioning using bivariate correlations. Scores for pre-mentalizing modes were negatively related to parents’ respect for autonomy, *p* = 0.04, and parents’ quality of assistance, *p* = 0.046. Certainty about mental states was also negatively related to parental sensitivity, i.e., stimulation of cognitive development, *p* = 0.009, and quality of assistance, *p* = 0.035. In contrast, parents who scored higher regarding interest and curiosity showed more supportive presence when interacting with their child, *p* = 0.017. Please note that all above descriptive statistical values are uncorrected.

**Table 2:** Intercorrelations between PRFQ scales, parental sensitivity scales and parental age

	Parental reflective functioning				Parental sensitivity		
	1	2	3	4	5	6	7
*Parental reflective functioning*							
1. Pre-mentalizing modes	1						
2. Certainty about mental states	− 0.060	1					
3. Interest and curiosity	− 0.122	0.158^+^	1				
*Parental sensitivity*							
4. Supportive presence	− 0.123	− 0.117	0.223*	1			
5. Respect for autonomy	− 0.192*	− 0.008	0.029	0.597**	1		
6. Stimulation of cognitive development	− 0.127	− 0.241**	0.008	0.646**	0.469**	1	
7. Quality of assistance	− 0.186*	− 0.197*	0.052	0.794**	0.553**	0.757**	1
8. Parental age	− 0.04	− 0.04	0.10	− 0.15	− 0.12	− 0.19*	− 0.17^+^

Uncorrected bivariate Pearson’s correlations, *N* = 115, ^+^*p* < 0.10, *p < 0.05, **p < 0.01

### Relationships Between Parental Attachment, Parental Reflective Functioning, and Parental Sensitivity

#### Parental Attachment and Parental Reflective Functioning

We conducted a MANOVA with three attachment classification groups and parental gender as the independent variables and the four parental sensitivity scales as the dependent variables (controlling for parental age) and provide all corresponding results in the supplement (Table S1). The analysis revealed a statistically significant difference in parental reflective functioning based on parental attachment, *F* (6, 212) = 2.197, *p* = 0.044; Wilk’s Λ = 0.886, partial η^2^ = 0.059, but no effect of parental gender or its interaction with attachment. Univariate comparisons showed significant differences between attachment groups for the interest and curiosity subscale only, *F* (2,108) = 3.31, *p* = 0.040, partial η^2^ = 0.058. FDR-corrected post hoc pairwise comparisons between attachment groups (see Table 3) revealed that dismissing parents scored significantly lower on interest and curiosity than securely attached parents, q = 0.036 (*p* = 0.012). Mean values suggested a similar pattern for parents classified as dismissing versus preoccupied, however, this difference did not reach significance.

**Table 3:** Means and standard deviations for PRFQ and parental sensitivity scales sorted by parental attachment classifications

	Parental attachment					
	Secure *n* = 63		Dismissing *n* = 33		Preoccupied *n* = 19	
	*M*	*SD*	*M*	*SD*	*M*	*SD*
*Parental reflective functioning*						
Pre-mentalizing modes	1.86	0.61	1.71	0.55	1.82	0.62
Certainty about mental states	4.04	1.09	4.34	1.14	3.92	1.16
Interest and curiosity	5.35a	0.78	4.87b	0.85	5.30ab	0.85
*Parental sensitivity*						
Respect for autonomy	3.95	1.10	3.64	1.10	4.11	1.41
Stimulation of cognitive development	3.38b	1.17	2.76c	0.83	4.00a	0.88
Quality of assistance	4.65a	1.10	4.00b	1.20	5.16a	1.07
Supportive presence	3.92a	1.29	3.21b	1.41	4.11a	1.37

*N* = 115, *M* = mean, *SD* = standard deviation, letter superscripts demonstrate significant (*p* < 0.05) differences between parental attachment groups on parental reflective function and parental sensitivity scales with scores marked a > b > c; note that differences between parental attachment groups regarding supportive presence remained significant (*p* < 0.10) after FDR corrections

#### Parental Attachment and Parental Sensitivity

Complete results of the MANOVA with three attachment classification groups and parental gender as the independent variables and the three parental reflective functioning scales as the dependent variables (controlling for parental age) can be found in the supplement (Table S2). There was a statistically significant difference in parental sensitivity based on parental attachment, *F* (8,212) = 2.450, *p* = 0.015; Wilk’s Λ = 0.837, partial η^2^ = 0.085, but no effect of parental gender or its interaction with attachment. Univariate comparisons of attachment groups revealed significant differences for stimulation of cognitive development, *F* (2,108) = 8.40, *p* < 0.001, partial η^2^ = 0.14, and quality of assistance, *F* (2,108) = 4.78, *p* = 0.010, partial η^2^ = 0.08, but only a marginally significant difference for supportive presence, *F* (2,108) = 2.91, *p* = 0.059, partial η^2^ = 0.051.

FDR-corrected post hoc comparisons revealed that dismissing parents scored generally lower than secure and preoccupied parents on these three scales as portrayed in Table 3. Regarding stimulation of cognitive development, dismissing parents scored lower than secure parents, *q* = 0.015 (*p* = 0.013), as well as lower than preoccupied parents, *q* = 0.003 (*p* = 0.001), and, preoccupied parents scored the highest with scores even above those of secure parents, *q* = 0.015 (*p* = 0.015). Regarding quality of assistance, dismissing parents scored lower than secure parents, *q* = 0.041 (*p* = 0.027), and lower than preoccupied parents, *q* = 0.012 (*p* = 0.004), while there was no difference between the secure and the preoccupied group. Finally, regarding supportive presence, dismissing parents scored marginally lower than secure parents, *q* = 0.081 (*p* = 0.031), as well as marginally lower than preoccupied parents, *q* = 0.081 (*p* = 0.054), while there was no difference between secure and preoccupied parents.

#### Parental Sensitivity and Parental Reflective Functioning

To further explore the role of parental gender in the relationship between parental reflective functioning and parental sensitivity, we calculated four regression analyses, one for each parental sensitivity scale as the outcome variable (for detailed results see supplement, Tables S3–S5). Neither the main effect of parental gender nor its interaction with parental reflective functioning was significant. However, the regression analysis results further confirmed and statistically solidified the correlational associations between parental reflective functioning and parental sensitivity reported above. For supportive presence, the PRFQ subscale interest and curiosity was a significant (*ß* = 0.20; *p* = 0.045) and certainty about mental states a marginally significant (*ß* = − 0.16; *p* = 0.099) predictor. For stimulation of cognitive development, parental age (*ß* = − 0.19.; *p* = 0.05) as well as the PRFQ subscale certainty about mental states (*ß* = − 0.27.; *p* = 0.005) were significant predictors. Quality of assistance was significantly predicted by the subscale certainty about mental states (*ß* = − 0.24.; *p* = 0.013) and on a marginally significant level predicted by the pre-mentalizing modes subscale (*ß* = − 0.18.; *p* = 0.06). Finally, respect for autonomy was marginally predicted by PRFQ pre-mentalizing subscale (*ß* = − 0.16.; *p* = 0.095), though the overall regression model was not significant.

### Exploratory Mediational Analyses Between Parental Attachment, Parental Reflective Functioning, and Parental Sensitivity.

So far, our results revealed specific patterns suggesting that parents classified as dismissing in the AAI systematically differed from those classified as secure (and partly from those classified as preoccupied). This is because parents classified as dismissing scored lower on the PRFQ subscale interest and curiosity as well as three parental sensitivity scales (i.e., supportive presence, stimulation of cognitive development, quality of assistance). Regarding the association between parental reflective functioning and parental sensitivity, our correlational analyses further found parents’ interest and curiosity to be solely associated with parents’ supportive presence.

**Fig. 1:**
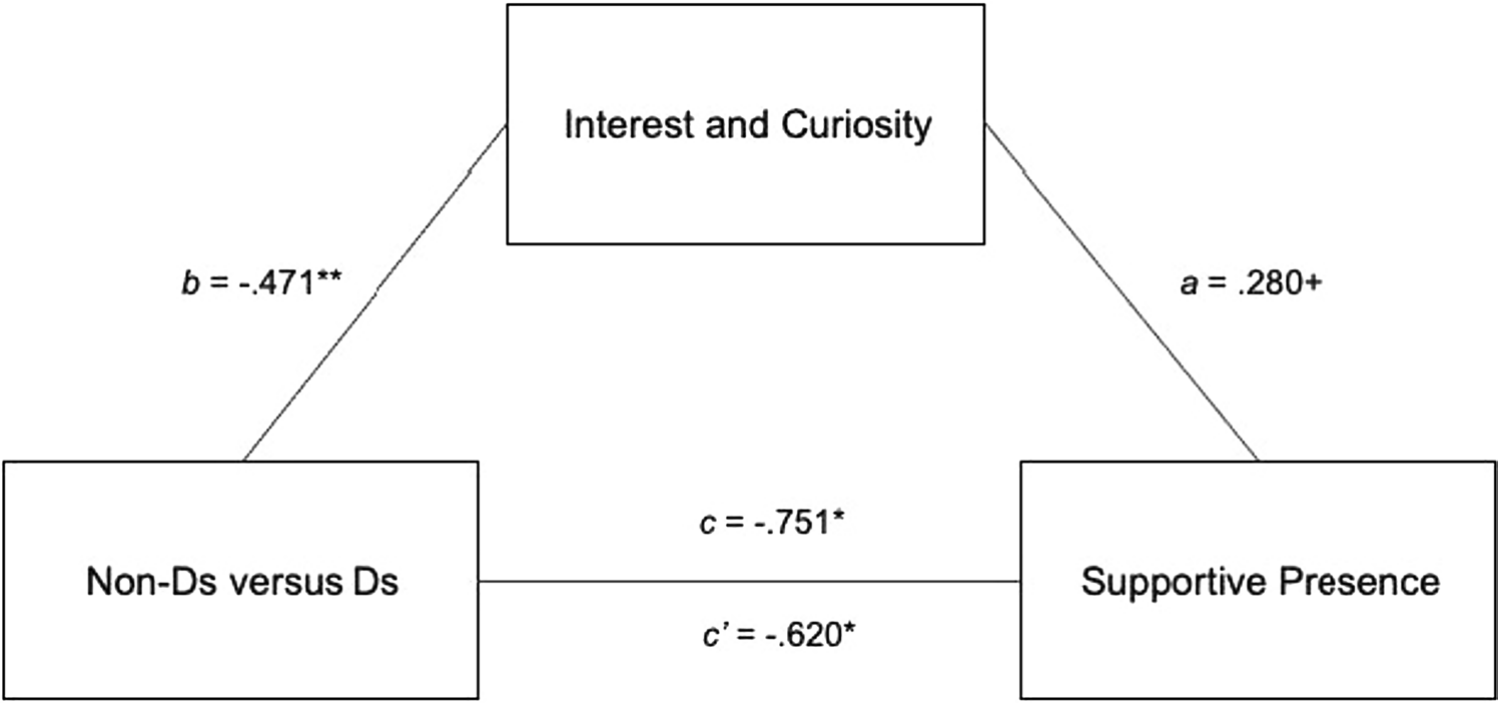
Mediation model for the relationship between parents’ dismissing status and supportive presence as mediated by parental interest and curiosity. *Ds* dismissing classification in the Adult Attachment Interview, *Non-Ds* secure and preoccupied classification in the Adult Attachment Interview, *N* = 115

In accordance with the above, we exploratively tested whether the negative relationship between parents’ dismissing status and parents’ supportive presence was mediated by decreased interest and curiosity found in dismissing parents in additional explorative data-driven analyses. For these mediation analyses, we coded the dichotomous variable parents’ dismissing status with 0 = Non-Ds (*n* = 82) and 1 = Ds (*n* = 33). The overall model (see Fig. 1) with parental interest and curiosity as the mediator was significant, *F (*2,112) = 5.48, *p* = 0.005, *R-sq* = 0.10. Dismissing status significantly predicted interest and curiosity, *B* = − 0.47, *se* = 0.17, *t* (113) = − 2.83, *p* = 0.006, which in turn predicted supportive presence on a marginal significance level, *B* = 0.28, *se* = 0.15, *t*(112) = 1.82, *p* = 0.07. The relationship between dismissing status and supportive presence was partially mediated by parents’ interest and curiosity, indirect effect *B* = − 0.13, 95% CI (− 0.347, − 0.005), direct effect, *B* = − 0.62, s*e* = 0.28, *t*(112) = − 2.20, *p* = 0.03.

When entering the covariate parental gender into the model, the reported links were still significant, direct effect, *B* = − 0.59, *se* = 0.28, *t* (111) = − 2.06, *p* = 0.04, however, the indirect effect of parental interest and curiosity as the mediator in the relation dismissing status and supportive presence weakened and became only marginally significant, *B* = − 0.103, 90% CI (− 0.256, − 0.003).

## Discussion

With our study we aimed to investigate the interplay between parental reflective functioning, attachment, and sensitivity. The points of added value comprise several methodological advancements. Above all, we want to highlight the use of gold standard methods assessing parental attachment representations (AAI) and parental sensitivity (observed parent–child interaction), which is valuable for research on parental reflective functioning in general, and particularly regarding a further validation of the PRFQ. Further methodological advancements include the focus on parents of preschool children and the inclusion of both mother–child and father–child dyads.

### Central Findings

Our central findings indicated significant relations between parental reflective functioning, attachment representations and sensitivity in both mothers and fathers, which particularly highlights the role of dismissing attachment in parenting on both cognitive (i.e., parental reflective functioning) as well as behavioral (i.e., sensitivity) levels. More precisely, parents classified as dismissing reported less interest and curiosity in their children’s mental state and scored lower on sensitivity during interaction than those classified as secure (and preoccupied). Furthermore, as predicted, parental reflective functioning was associated with parental sensitivity. Interestingly, as dismissing attachment status appeared to be crucial for parental reflective functioning as well as sensitivity in our sample, we exploratively tested a mediational model. Our findings tentatively suggest that parents’ interest and curiosity partially mediate the relationship between dismissing attachment status and supportive presence. Finally, we found the PRFQ to be a valid measure of parental reflective functioning that in both mothers and fathers is similarly related to attachment and sensitivity.

### Parental Attachment Representations and Parental Reflective Functioning

As expected, we found a positive association between parents’ secure attachment representation and self-reported parental reflective functioning, but this was only the case for the interest and curiosity subscale. Interestingly, securely attached parents reported increased interest and curiosity in their children’s mental state as compared to those classified as dismissing, but did not differ from those classified as preoccupied in the AAI. While there are no other studies relating parental reflective functioning derived from the PRFQ to parental attachment classified with the AAI, our findings are mostly in line with those emerging from self-report measures of attachment. For example, parents’ interest and curiosity in the PRFQ was negatively associated with avoidance in the ECR-R [18] and positively associated with security in the ASQ [27]. However, Luyten et al. [3] found interest and curiosity to be unrelated to self-reported attachment in the ECR-R. The pattern of our findings regarding differences between both insecure attachment classifications are supported by Milligan et al. [46], who found preoccupied mothers to use more emotion related infant-directed mind-mindedness than mothers classified as dismissing. Given that attachment classifications are closely linked to certain emotion regulation strategies [47] our results indicate that—at least in low stressor contexts—the secure and preoccupied attachment patterns may share some common qualities. Still, further research is needed to address the question whether preoccupation may indeed lead to rather dysfunctional hyper-reflection, which may especially come to light in more negative or stressful contexts [46, 48].

According to attachment theory, the observed negative association between interest and curiosity and dismissing attachment (or avoidance) appears valid. In contrast to the preoccupied or secure attachment representations, dismissing attachment is characterized by lower activation the attachment system and diminished attention to attachment-related experiences and cognitions [49–52]. Decreased interest and curiosity in children’s mental state may be a consequence of dismissing parents’ tendency to suppress and withdraw from emotional or attachment relevant information.

Notably, previous research using the AAI and narrative measures to assess parental reflective functioning found that not only dismissing, but also preoccupied parental attachment leads to mentalizing deficits [20]. Still, the authors suggest that there are “ways of being reflective that are more avoidant and dismissing and others that are more preoccupied” ([20], p. 295). Possibly the interest and curiosity subscale of the PRFQ is more suitable to capture aspects of parental reflective functioning that relate to deactivation of the attachment system as characteristic for dismissing attachment while not differentiating between secure and preoccupied attachment. The latter attachment classifications probably both share a fundamental interest in mental states. It is conceivable that certain aspects of mentalizing (e.g., envisioning children’s mental state, understanding intentions and feelings underlying child behavior) that may differentiate between secure and preoccupied attachment rather reflect in the PRFQ pre-mentalizing modes. However, we did not find levels of pre-mentalizing modes to vary as a function of parental attachment in our sample. Further studies with larger and more varied samples are needed to confirm specific associations between different attachment representations and parental reflective functioning in the PRFQ.

### Parental Attachment Representations and Sensitivity

As expected, secure parental attachment was associated with higher levels of parental sensitivity during parent–child interaction, but only when comparing secure and dismissing attachment categories. More precisely, parents classified as dismissing scored significantly lower than those classified as secure regarding stimulation of cognitive development as well as quality of assistance and marginally lower regarding supportive presence. These findings correspond well with previous studies examining parental attachment representations and parental sensitivity, as altered caregiving behavior in parents with insecure attachment representations is a widely reported finding in infant and preschool studies [35, 53]. In our sample, parents classified as dismissing scored lower not only than secure but also than preoccupied parents even though they were both assigned an insecure classification. In fact, in our study, preoccupied parents were comparable to secure parents regarding their parenting behavior, which supports findings by Pederson et al. [54]. Interestingly, Whipple et al. [55] found the dismissing and preoccupied dimension in the AAI to be individually related to specific parenting behaviors.

Our findings regarding attachment-specific parenting behavior reflect the aforementioned pattern we found for parental reflective functioning and corroborate our assumption of a specific mechanism inherent to dismissing rather than preoccupied parents that may negatively affect central aspects of caregiving. Taken together, our results support a characteristic of dismissing parents that was referred to as a “low-investment” ([56], p. 361) or “cool and remote” parenting style ([57], p. 1283). Our data further highlight the importance of differentiating between insecure-dismissing and insecure-preoccupied classifications, which theoretically refer to rather diametrical than comparable mental states.

It should be noted, however, that we observed parenting in a laboratory situation in a low-risk sample. Possibly, preoccupied attachment may not hinder parents to interact with their child on a functional level comparable to securely attached parents. In contrast to withdrawal tendencies characteristic for dismissing attachment, preoccupation may be linked to a general interest in children’s emotional needs and an openness necessary for vivid dyadic interactions. Higher levels of induced stress and negative emotional contexts may, however, challenge preoccupied parents’ capacities [46]. Further research is needed to disentangle relations between preoccupation, parental reflective functioning and parental sensitivity in more naturalistic and emotionally stressful situations.

### Parental Reflective Functioning and Sensitivity

Confirming our hypothesis, we found parental reflective functioning to be predictive of parental sensitivity during the interaction. More precisely, certainty about mental states was negatively associated with quality of assistance as well as stimulation of cognitive development and marginally negatively associated with supportive presence. According to theory, recognizing that one cannot know everything about another’s feelings and intentions (i.e., “opacity of mental states”) is one of the key features of genuine reflective functioning [58]. It captures parents’ ability to understand children’s mental states as being ultimately opaque and distinctive of their own mental states [20]. In contrast to self-report-studies that assume moderate levels of certainty about mental states to be most adaptive for parenting [3], we found parents who report to be very certain about their child’s mental states to score lower on parental sensitivity and that this association was rather linear than u-shaped. We conclude that higher scores on certainty about mental states may thus be indicative of hyper-mentalizing tendencies [3] and that they may interfere with being attentive to children’s signals during the interaction. For example, a parent assuming their child is confident given a certain task may pay less attention to and miss subtle signals of discomfort. As a consequence, this mismatch or emotional invalidation may negatively impact the child’s own emotional awareness [59].

Similarly, we also found pre-mentalizing modes to be indicative of less optimal parenting. Thus, at trend-level, parents showing higher levels of pre-mentalizing modes showed less respect for children’s autonomy and a decreased level of quality of assistance during the interaction. This may impede children to tackle the task themselves and to experience associated self-efficacy while being adequately guided by an adult. Our findings confirm and extend former research reporting associations between parental pre-mentalizing modes and observed parental sensitivity [32] as well as self-reported deficits in parenting (e.g., [3]. In contrast, interest and curiosity was positively and solely related to supportive presence. This is in line with findings from Luyten and colleagues who reported interest and curiosity to be positively associated with self-reported emotional availability [3]. Taken together, the associations between self-reported parental reflective functioning and observed parental sensitivity support prior findings suggesting that parental reflective functioning plays a crucial role for parenting quality [14, 31, 60].

### Exploratory Mediation Analyses Between Parental Attachment Representations, Parental Reflective Functioning, and Sensitivity

Finally, we assumed that parental reflective functioning would mediate the association between parental attachment representation and sensitivity. Exploratory mediation analyses suggested that the relationship between dismissing attachment status and supportive presence was partially mediated by parents’ interest and curiosity. This finding may imply that parental reflective functioning may play an important role for understanding the link between parental attachment representation and sensitivity and thus the intergenerational transmission of attachment (also see [20]). Accordingly, our findings suggest that interest and curiosity as a motivational aspect in parents’ cognition could be a valuable focus of interventions. This may be especially true for more dismissing parents, who—possibly due to their own rearing background—tend to withdraw from attachment relevant content and display restrained emotional investment. Detailed practical implications of our findings are summarized in a corresponding paragraph below.

### Findings Regarding the PRFQ Validation

Our study altogether supports existing research that found the PRFQ to be a valid and economic self-report measure for the assessment of parental reflective functioning [3] with the caveat that certain components (pre-mentalizing modes) may not be captured adequately in this German version of the self-report. Still, our findings generally confirmed its construct validity by including behavioral and narrative measures of parental sensitivity and attachment. The PRFQ exhibited moderate to good internal consistency on both the interest and curiosity and the certainty about mental states subscales. However, similar to another recent study using the German version of the PRFQ, internal consistency of the pre-mentalizing modes subscale was rather low (see [32]). Cronbach’s alpha may be affected by the low number of items [61] and very low variances of single items in this scale. As mentioned, the scale was left-skewed with a mean of 1.81 (*SD* = 0.59). Interestingly, including a large sample of mothers and fathers of children 3–10 years, Pazzagli et al. [27] found comparably low mean values (*M* = 1.79, *SD* = 0.85) in parents of preschool children that significantly differed from pre-mentalizing modes in parents of older children. The authors argue this may be due to the fact that younger children’s motives and needs may be more basic and easier to determine leaving less room for interpretation. However, given the fact that Krink et al. [32] as the only other study using the German version of the PRFQ also found low internal consistencies of the pre-mentalizing modes subscale, a revision may be considered. While our findings provide valuable information on the useability of the PRFQ in normative samples in general -and the German version in particular- further studies with different age groups and more heterogeneous samples are needed to strengthen this empirical evidence. A factor analysis confirmed the structure with three independent factors, except for a marginally positive relationship between interest and curiosity in mental states and certainty about mental states. Notably, in our sample one item (“I believe there is no point in trying to guess what my child feels”) loaded on pre-mentalizing modes instead of interest and curiosity. This inconsistency may partly derive from the German translation and ambiguous wording regarding this item, which has also been noticed by Krink et al. [32]. Also, deleting the item from the interest and curiosity scale increases Cronbach’s alpha to 0.65. Finally, corresponding with other studies, in our sample mothers scored higher on interest and curiosity in mental states than fathers [27].

### Strengths and Limitations

With our study we shed further light on possible precursors and effects of parental reflective functioning, which has repeatedly been found to play a key role in parenting. The distinctive nature of our study lies in our multi-modal approach encompassing gold standard measures of parental attachment and behavioral observations of parental sensitivity in a sizable sample of both fathers and mothers and their preschool-aged children. Nevertheless, some limitations should be addressed. First, due to the aforementioned relatively low internal consistency of the pre-mentalizing modes subscale, corresponding findings should be interpreted cautiously. Also, some effects were only marginally significant and need to be interpreted with caution. Second, a methodological limitation refers to our mediational approach in the exploratory analyses. To draw conclusions regarding the casualty of effects, further research including longitudinal data is needed. However, by theory we assume parental attachment to precede parenting behavior, which is also supported by numerous longitudinal studies (for review see van Ijzendoorn [35]). Third, there are some aspects regarding our study design that restrict the generalization of our findings. For example, we only observed parental behavior during mild stressor situations that were structured and limited to ten minutes. Also, in our study, mothers and fathers constituted a low-risk sample of White European origin. To generalize our findings, future studies need to address our research questions in a more diverse population including parents and children from different cultural and medical backgrounds.

## Implications for Prevention and Intervention Programs

In prevention and intervention programs targeting sensitive parenting, parental reflective functioning and mentalizing appear to be key factors that need to be addressed, ideally by taking into account parents’ own attachment experiences and encouraging introspection. Here, parenting training programs may especially benefit from fostering interest and curiosity in children's unique mental states while also pointing to the opacity of children’s inner world, e.g., by using video-based procedures [62]. Attachment-based dyadic interventions focus on positive aspects of parent–child interactions in which parents adequately respond to their children’s needs and point out their children’s corresponding reaction or delight (for a review, see [62]). Observing these valuable moments can be very encouraging for parents and potentially have a dramatic impact on their self-efficacy. For example, the ABC (Attachment and Biobehavioral Catch-Up) intervention includes sessions in which parents are instructed to “follow the child’s lead” and encouraged to be curious and interested in their child [63]. Video-feedback may thereby vitally contribute to positive outcomes of family programs by helping parents to gain insights in their child’s inner world (for a review, see [62]).

Furthermore, Steele and colleagues summarize that observing interactions in videos serves “as a powerful catalyst for reflective functioning and updating one’s frame of reference for how to think, feel and behave with one’s child” ([64], p. 402). Indeed, video-feedback interventions enable the detection of children’s signals on a micro-analytic level and stimulate parents' reflection about the fitting of their reaction to the child’s current inner state. Also, possible misinterpretations regarding children’s actions and intentions can be discussed with trainers and evaluated in a calm and appreciative setting. In sum, providing an atmosphere that encourages parents to take a step back and reflect upon interactions with their children on a metacognitive level can strengthen parents' co-regulatory capabilities and promote healthy development of the parent–child attachment relationship (for further information on how to improve parental reflective functioning [14, 65]).

## Concluding Remarks

In summary, our study confirms and extends research on parental reflective functioning by linking the capacity to think about children’s internal states to parents’ own attachment history in both mothers and fathers. We further show its positive impact on sensitive parenting behavior in the preschool years. Our findings especially highlight the crucial role of dismissing attachment status. More precisely, we found this strategy to be linked to withdrawal in parenting on both cognitive (i.e., decreased reflective functioning) and behavioral (i.e., decreased sensitivity) levels.

Our findings emphasize that both mothers’ and fathers’ parenting competencies are similarly related to specific interrelated factors (i.e., parental reflective functioning and attachment) possibly representing an intergenerational mechanism. As suggested, parental reflective functioning may be one of the mediating factors between parents’ own attachment history and children’s attachment [20, 66]. Also, parental sensitivity was found to mediate the impact of parental reflective functioning on infant attachment [6, 7]. Our findings extend these ideas by further revealing a link between parental attachment, parental reflective functioning and sensitivity, which appears to be comparably present in both mothers and fathers.

Considering the importance of parent–child attachment to further developmental adaptation [4] and mental health [5] it is crucial to further expand our knowledge on factors involved in intergenerational mechanisms, i.e. parental reflective functioning and put it into practice. Notably, this should also include the aim to gain more insight about different operationalizations and the specific dimensions of parental reflective functioning they may tap into.

## Summary

Parental reflective functioning refers to parents’ capacity to navigate their children’s inner world and hold their children’s mental states in mind and is an essential ingredient of parenting. It is assumed to positively affect children’s healthy mental development by supporting children in gaining access to their own emotions and intentions and making sense of their own and others’ behaviors. With the PRFQ, researchers and practitioners have a brief, multidimensional assessment of parental reflective functioning at their disposal to economically screen parents regarding this capacity. Parental reflective functioning is further thought to provide a missing link between parents' own attachment histories and their parenting behaviors. To take these ideas forward, the current study aimed to investigate the interplay between parents’ attachment representations as well as self-reported parental reflective functioning and behaviorally coded sensitivity during parent–child interaction. Participants were a large sample of parents, both mothers and fathers, with their preschool aged children. Applying a multi-modal approach, we implemented the AAI, the German version of the PRFQ, and behavioral observations of parental sensitivity during parent–child problem-solving. Our findings suggest that parents classified as insecure dismissing in the AAI may be particularly prone to deficiencies in parental reflective functioning, as indicated by decreased PRFQ interest and curiosity in child mental states subscale scores. Additionally, this group of parents showed less sensitive parenting behavior as compared to the secure and insecure preoccupied groups. In exploratory mediation analyses, we further found that the relationship between parents’ dismissing attachment and decreased behavioral sensitivity may be partially mediated by decreased self-reported interest and curiosity (PRFQ). Notably, our results were comparable between mothers and fathers as well as independent of child biological sex. Our findings highlight the crucial role parental reflective functioning plays for the quality of parenting behavior. They provide further evidence for a link between parental reflective functioning and attachment representations, which suggests an intergenerational mechanism. Our findings are particularly relevant for practitioners working with families by emphasizing the need to target parental reflective functioning in prevention and intervention programs (i.e., raising parents’ interest and curiosity in their children’s mental states). To do so, video-based interventions and educational elements conveying the importance of parents’ own attachment histories may be particularly relevant to illustrate the tight links between parental reflective functioning and parenting behavior and their meaning for children’s healthy development. Finally, our findings serve as a further validation of the PRFQ given the caveat that the pre-mentalizing modes subscale may need further revision in the German version.

## Supplementary Information

Below is the link to the electronic supplementary material.

Supplementary file1 (DOCX 41 KB)

## Data Availability

At the time the research was undertaken, not all participants provided explicit written permission by means of informed consent for their individual raw data to be made publicly available. Therefore, the complete raw data cannot be openly shared. Upon receipt of a reasonable request and guaranteed data anonymity (i.e., exclusion of any potentially identifiable information), restricted access to the raw data may nonetheless be granted.

## Materials Availability

The German version of the PRFQ is available for download here: https://www.ucl.ac.uk/psychoanalysis/research/parental-reflective-functioning-questionnaire-prfq. The German version of the AAI is published in Gloger-Tippelt [38]. Researchers who are interested in the employed sensitivity scales are advised to contact the authors.
