# Supplementary material for: Precursors and Effects of Self-reported Parental Reflective Functioning: Links to Parental Attachment Representations and Behavioral Sensitivity
Source: Child Psychiatry Hum Dev. 2024 Feb 7;56(6):1598–613. doi: 10.1007/s10578-023-01654-2 (PMC12628443; doi:10.1007/s10578-023-01654-2)
Supplement: Supplementary file 1 — Supplementary file1 (DOCX 41 KB) [file 10578_2023_1654_MOESM1_ESM.docx]

**Supplementary Material**

**Table S1**

*Results of the MANOVA examining the associations between parental attachment and sensitivity (controlling for parental gender and age).*

| **Multivariate tests** | | | | | | | |
| --- | --- | --- | --- | --- | --- | --- | --- |
| Effect | | Wilk’s lambda | *F* | Hypothesis *df* | Error *df* | Sig. | Partial eta squared |
| Parental age |  | .969 | .839 | 4.000 | 105.000 | .504 | .031 |
| Attachment 3-way |  | .837 | 2.450 | 8.000 | 210.000 | .**015** | .085 |
| Parental gender |  | .949 | 1.412 | 4.000 | 105.000 | .235 | .051 |
| Parental gender *  attachment 3-way |  | .910 | 1.261 | 8.000 | 210.000 | .266 | .046 |
|  | | | | | | | |
|  | | | | | | | |

| **Tests of between-subjects effects** | | | | | | | |
| --- | --- | --- | --- | --- | --- | --- | --- |
|  | Dependent variable | Type III sum of squares | *df* | Mean square | *F* | Sig. | Partial eta squared |
| Parental age | Supportive presence | 3.094 | 1 | 3.094 | 1.718 | .193 | .016 |
|  | Respect for autonomy | 1.610 | 1 | 1.610 | 1.242 | .268 | .011 |
|  | Quality of assistance | 2.900 | 1 | 2.900 | 2.339 | .129 | .021 |
|  | Stimulation of cognitive development | 3.498 | 1 | 3.498 | 3.304 | .072 | .030 |
| Attachment 3-way | Supportive presence | 10.480 | 2 | 5.240 | 2.909 | **.059** | .051 |
|  | Respect for autonomy | 1.350 | 2 | .675 | .521 | .595 | .010 |
|  | Quality of assistance | 11.847 | 2 | 5.924 | 4.777 | **.010** | .081 |
|  | Stimulation of cognitive development | 17.783 | 2 | 8.891 | 8.398 | **<.001** | .135 |
| Parental gender | Supportive presence | .827 | 1 | .827 | .459 | .499 | .004 |
|  | Respect for autonomy | 3.460 | 1 | 3.460 | 2.670 | .105 | .024 |
|  | Quality of assistance | 1.840 | 1 | 1.840 | 1.484 | .226 | .014 |
|  | Stimulation of cognitive development | .019 | 1 | .019 | .018 | .894 | .000 |
| Attachment 3-way * parental gender | Supportive presence | .130 | 2 | .065 | .036 | .965 | .001 |
|  | Respect for autonomy | 2.870 | 2 | 1.435 | 1.107 | .334 | .020 |
|  | Quality of assistance | 1.574 | 2 | .787 | .635 | .532 | .012 |
|  | Stimulation of cognitive development | 2.122 | 2 | 1.061 | 1.002 | .370 | .018 |
| Error | Supportive presence | 194.517 | 108 | 1.801 |  |  |  |
|  | Respect for autonomy | 139.955 | 108 | 1.296 |  |  |  |
|  | Quality of assistance | 133.924 | 108 | 1.240 |  |  |  |
|  | Stimulation of cognitive development | 114.340 | 108 | 1.059 |  |  |  |
| *Note:* *N* = 115 | | | | | | | |
|  | | | | | | | |
| **Table S2** | | | | | | | |
| *Results of the MANOVA examining associations between parental attachment and reflective functioning (Controlling for parental gender and age).* | | | | | | | |

| **Multivariate tests** | | | | | | | |
| --- | --- | --- | --- | --- | --- | --- | --- |
| Effect | | Wilk’s Lambda | *F* | Hypothesis *df* | Error *df* | Sig. | partial eta squared |
| Parental age |  | .973 | .993^b^ | 3.000 | 106.000 | .399 | .027 |
| Attachment 3-way |  | .886 | 2.197^b^ | 6.000 | 212.000 | **.044** | .059 |
| Parental gender |  | .952 | 1.767^b^ | 3.000 | 106.000 | .158 | .048 |
| Attachment 3-way *  parental gender |  | .981 | .346^b^ | 6.000 | 212.000 | .912 | .010 |
| *Note:* *N* = 115 | | | | | | | |
|  | | | | | | | |
|  | | | | | | | |

| **Tests of between-subjects effects** | | | | | | | |
| --- | --- | --- | --- | --- | --- | --- | --- |
|  | Dependent variable | Type III Sum of squares | *df* | Mean square | *F* | Sig. | Partial eta squared |
| Parental age | Prementalizing modes | .009 | 1 | .009 | .489 | .486 | .005 |
|  | Certainty about mental states | .082 | 1 | .082 | .064 | .801 | .001 |
|  | Interest and curiosity | 1.769 | 1 | 1.769 | 2.769 | .099 | .025 |
| Attachment 3-way | Prementalizing modes | .035 | 2 | .018 | .935 | .396 | .017 |
|  | Certainty about mental states | 3.440 | 2 | 1.720 | 1.345 | .265 | .024 |
|  | Interest and curiosity | 4.229 | 2 | 2.114 | 3.311 | **.040** | .058 |
| Parental gender | Prementalizing modes | .036 | 1 | .036 | 1.945 | .166 | .018 |
|  | Certainty about mental states | .576 | 1 | .576 | .450 | .504 | .004 |
|  | Interest and curiosity | 2.547 | 1 | 2.547 | 3.988 | **.048** | .036 |
| Attachment 3-way *  parental gender | Prementalizing modes | .003 | 2 | .002 | .085 | .918 | .002 |
|  | Certainty about mental states | 1.430 | 2 | .715 | .559 | .573 | .010 |
|  | Interest and curiosity | .482 | 2 | .241 | .378 | .686 | .007 |
| Error | Prementalizing modes | 2.024 | 108 | .019 |  |  |  |
|  | Certainty about mental states | 138.146 | 108 | 1.279 |  |  |  |
|  | Interest and curiosity | 68.968 | 108 | .639 |  |  |  |
| *Note:* *N* = 115 | | | | | | | |
|  | | | | | | | |
|  | | | | | | | |

**Table S3**

*Multiple hierarchical regression analyses for variables predicting parental sensitivity*

| **1 Regression analysis for supportive presence** | *B* | *SE B* | *ß* | *t* | *p* |
| --- | --- | --- | --- | --- | --- |
| Step 1 |  |  |  |  |  |
| Parental gender | -.33 | 2.01 | -.12 | -.16 | .870 |
| Step 2 |  |  |  |  |  |
| Prementalizing modes | -.16 | .22 | -.07 | -.76 | .450 |
| Certainty about mental states | -.19 | .12 | -.16 | -1.68 | **.099** |
| Interest and curiosity | .32 | .16 | .20 | 2.04 | **.045** |
| Step 3 |  |  |  |  |  |
| sex * prementalizing modes | -.22 | .22 | -.30 | -1.00 | .321 |
| sex * certainty about mental states | .15 | .12 | .45 | 1.24 | .217 |
| sex * interest and curiosity | -.05 | .16 | -.17 | -.28 | .780 |
| **2 Regression analysis for respect for autonomy** | *B* | *SE B* | *ß* | *t* | *p* |
| Step 1 |  |  |  |  |  |
| Parental gender | .91 | 1.71 | .40 | .53 | .590 |
| Step 2 |  |  |  |  |  |
| Prementalizing modes | -.31 | .18 | -.16 | -1.68 | **.095** |
| Certainty about mental states | -.04 | .10 | -.04 | -.42 | .67 |
| Interest and curiosity | -.06 | .14 | -.04 | -.43 | .67 |
| Step 3 |  |  |  |  |  |
| sex * prementalizing modes | -.21 | .184 | -.35 | -1.14 | .26 |
| sex * certainty about mental states | .07 | .099 | .28 | .75 | .45 |
| sex * interest and curiosity | .13 | .1359 | .62 | .99 | .32 |
| **3 Regression analysis for stimulation of cognitive development** | *B* | *SE B* | *ß* | *t* | *p* |
| Step 1 |  |  |  |  |  |
| Parental age | -.04 | .02 | -.19 | -1.97 | **.05** |
| Parental gender | .50 | 1.62 | .23 | .31 | .76 |
| Step 2 |  |  |  |  |  |
| Prementalizing modes | -.23 | .17 | -.12 | -1.34 | .19 |
| Certainty about mental states | -.27 | .09 | -.27 | -2.84 | **.005** |
| Interest and curiosity | .02 | .13 | .015 | .15 | .881 |
| Step 3 |  |  |  |  |  |
| sex * prementalizing modes | -.15 | .17 | -.26 | -.85 | .398 |
| sex * certainty about mental states | .03 | .09 | .12 | .34 | .738 |
| sex * interest and curiosity | .10 | .13 | .46 | .75 | .455 |
| **4 Regression analysis for quality of assistance** | *B* | *SE B* | *ß* | *t* | *p* |
| Step 1 |  |  |  |  |  |
| Parental gender | .47 | 1.72 | .20 | .27 | .785 |
| Step 2 |  |  |  |  |  |
| Prementalizing modes | -.35 | .19 | -.18 | -1.91 | **.06** |
| Certainty about mental states | -.25 | .10 | -.24 | -2.52 | **.013** |
| Interest and curiosity | .03 | .14 | .02 | .19 | .85 |
| Step 3 |  |  |  |  |  |
| sex * prementalizing modes | -.13 | .19 | -.22 | -.73 | .470 |
| sex * certainty about mental states | -.03 | .10 | -.10 | -.26 | .795 |
| sex * interest and curiosity | .15 | .14 | .69 | 1.13 | .26 |

*Note.* *N* = 115. ^+^*p* < .10. ^*^*p* < .05. ^**^*p* < .01.;

^1^*R^2^* = .11; *adjusted R²* = .06; *F*(7) = 1.96; *p* = .067

^2^*R^2^* = .09; *adjusted R²* = .03; *F*(7) = 1.41; *p* = .208

^3^*R²* = .11; *adjusted R^2^* = .05; *F*(7) = 1.85; *p* = .086

^4^*R²* = .13; *adjusted R^2^* = .07; *F*(7) = 2.24; *p* = .036
